# Bidirectional, Daily Temporal Associations between Sleep and Physical Activity in Adolescents

**DOI:** 10.1038/s41598-019-44059-9

**Published:** 2019-05-22

**Authors:** Lindsay Master, Russell T. Nye, Soomi Lee, Nicole G. Nahmod, Sara Mariani, Lauren Hale, Orfeu M. Buxton

**Affiliations:** 1 Department of Biobehavioral Health, Pennsylvania State University, University Park, PA USA; 2 School of Aging Studies, University of South Florida, Tampa, FL USA; 3 Division of Sleep and Circadian Disorders, Brigham and Women’s Hospital, Boston, MA USA; 4 Division of Sleep Medicine, Harvard Medical School, Boston, MA USA; 5 Program in Public Health; Department of Family, Population and Preventive Medicine, Stony Brook University, Stony Brook, NY USA; 6 Department of Social and Behavioral Sciences, Harvard Chan School of Public Health, Boston, MA USA

**Keywords:** Risk factors, Neurology, Predictive markers

## Abstract

This study evaluated the daily, temporal associations between sleep and daytime physical activity and sedentary behavior among adolescents from the Fragile Families & Child Wellbeing Study. A sub-sample of the cohort at age 15 (N = 417) wore actigraphy monitors for one week during the school year from which we derived daily minutes in sedentary and moderate-to-vigorous physical activity (MVPA) and nighttime sleep measures. Multilevel models tested temporal associations of nightly sleep onset, offset, duration, and sleep maintenance efficiency, with daily MVPA and sedentary behavior. More MVPA than an individual’s average was associated with earlier sleep onset (p < 0.0001), longer duration (p = 0.03), and higher sleep maintenance efficiency (p < 0.0001). On days with more sedentary behavior than an individual’s average, sleep onset and offset were delayed (p < 0.0001), duration was shorter (p < 0.0001), and sleep maintenance efficiency was higher (p = 0.0005). Conversely, nights with earlier sleep onset predicted more next-day sedentary behavior (p < 0.0001), and nights with later sleep offset and longer sleep duration were associated with less MVPA (p < 0.0001) and less sedentary time (p < 0.0001, p = 0.004) the next day. These bidirectional associations between sleep and physical activity suggest that promoting MVPA may help to elicit earlier bedtimes, lengthen sleep duration, and increase sleep efficiency, critical for healthy adolescent development.

## Introduction

Adolescence is a critical developmental period, during which adequate sleep is important for optimal physical and behavioral maturation. Adolescents who regularly sleep 8–10 hours per night, as recommended by sleep and pediatric experts^1–3^, perform better in the classroom^4^, are more physically active^5^, and have lower rates of obesity^6^. Recent estimates indicate that 73% of adolescents report sleeping less than the necessary 8 hours, which is often attributed to extrinsic factors including early school start times^7^ misaligned with age-specific phase shifts towards later circadian preference (i.e., chronotype)^8^. This alarming rate of sleep insufficiency among adolescents warrants the exploration of behaviors that adolescents can modify to promote and protect their sleep health.

A recent meta-analysis concluded that individuals who participate in regular physical activity (i.e., months and years) are more likely to have adequate sleep duration, show improved sleep continuity, and experience less frequent daytime sleepiness^9^. A separate review article of studies from multiple age groups on sleep after an exercise intervention indicated that days with physical activity were typically followed by nights with better sleep quality, indicated by shortened sleep onset latency and reduced wake after sleep onset^10^. Findings in adolescent samples are consistent. In a national sample of high school students, those who reported 60 minutes or more of daily physical activity had higher odds of sufficient sleep duration compared to those who were active for less than 60 minutes^11^, suggesting that duration of physical activity is important in relation to sleep. Additionally, a recent randomized-control trial found that student participation in a 3-week running group (vs. a group seated during this time) significantly improved subjective sleep quality, shortened objective sleep onset latency, improved mood, reduced daytime sleepiness, and increased proportion of slow-wave sleep^12^.

More moderate-to-vigorous physical activity (MVPA) and less sedentary behavior are associated with longer and better quality sleep, but there is lack of research considering effects of both MVPA *and* sedentary behavior on multiple dimensions of sleep (beyond sleep duration). The relationships between sleep and physical activity are likely bidirectional, but research examining the temporal associations between these two health behaviors using multiple days of actigraphy data in adolescents is limited. This study aims to fill the gaps in prior research by examining the associations between time spent in MVPA and sedentary behavior and sleep outcomes (i.e., duration, sleep maintenance efficiency, timing) at the daily level among adolescents in their naturalistic environments.

**Figure 1:**
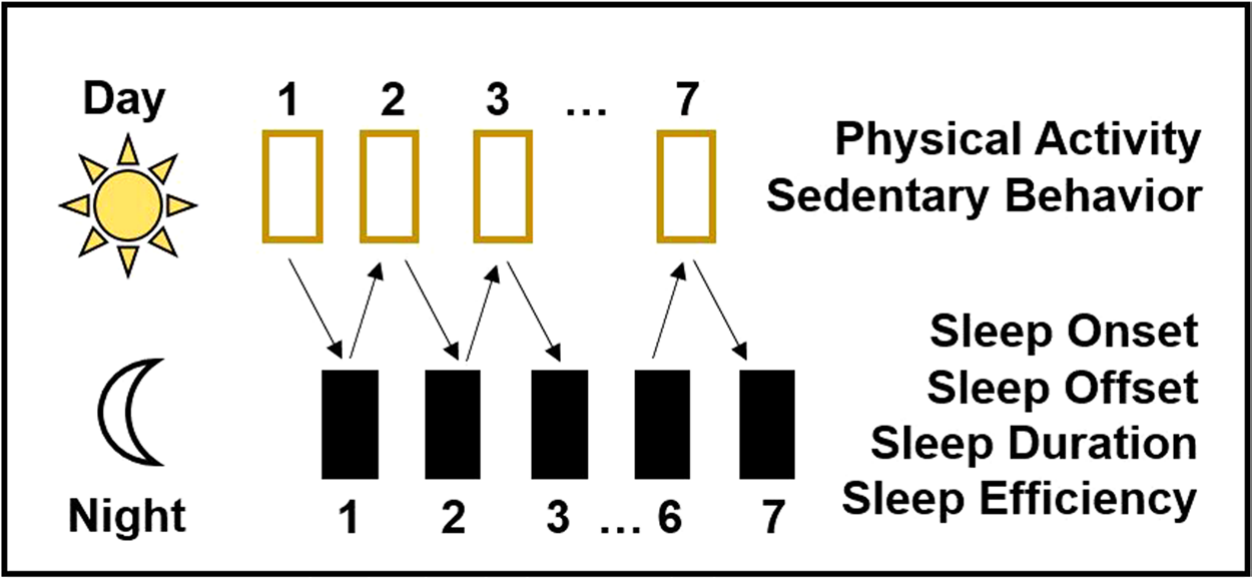
Study Design.

This micro-longitudinal observational study examined the temporal associations of objectively measured sleep and physical activity in a large, diverse cohort of adolescents across one week (Fig. 1). Specifically, we first examined daytime physical activity measures in relation to the timing, quality, and quantity of sleep that night, and then also examined nighttime sleep in relation to the physical activity the next day. We hypothesized that on days when adolescents were more physically active than their individual average (i.e., more MVPA, less sedentary behavior), they would have an earlier sleep onset, longer sleep duration, and higher sleep maintenance efficiency that night. In the opposite temporal direction, we hypothesized that adolescents who fell asleep earlier, slept longer, and had more efficient sleep during the night would spend more time in MVPA and less time being sedentary the following day. Ultimately, our findings will serve to better elucidate potential reciprocal associations between sleep and physical activity, and how physical activity may optimize adolescent sleep health.

## Results

### Descriptive statistics

Table 1 describes the characteristics of our study participants. Approximately half (47%) were males; 43% were Black, non-Hispanic, 28% were Hispanic, and 14% were multiracial or other race. Participants’ average age was 15.5 years old (SD = 0.6). The sample is largely healthy. The mean body mass index (BMI) percentile was 73.4 (SD = 25.1), which is heavier than the population mean, but not overweight (85^th^ percentile). More than half of participants had a BMI that was considered to be within the ‘normal’ range (54% healthy range, 21% overweight, 24% obese, and 2% below healthy range). Of individuals in the sample, 30% report Excellent health, 37% report Very Good health, 24% report Good health, 8% report Fair health, and <1% report Poor health.

**Table 1:** Participant Characteristics and Descriptive Statistics.

*Sociodemographic and Health Covariates*	Participants (N = 417)
	M or % (SD)
**Sex (%)**	
Male	47%
Female	53%
**Race (%)**	
White, non-Hispanic	15%
Black, non-Hispanic	43%
Hispanic	28%
Other	14%
**Mother’s Education (%)**	
Less than High School	18%
High School or Equivalent	19%
Some College/Technical School	48%
College or Graduate School	15%
**Family Structure (%)**	
Biological Mother + Biological Father	30%
Other Living Arrangements	9%
Biological Mother + New Partner	24%
Biological Mother Only	37%
**Income to Poverty Threshold (%)**	
<49%	13%
50–99%	17%
100–199%	28%
200–299%	14%
>=300%	28%
**Child Age** (**years**)	15.5 (0.6)
**Body Mass Index Age Percentile** (**%**)	73.4 (25.1)
***Main Variables***	
Sleep Onset (time, hh:mm)	12:04 AM (1:19)
Sleep Offset (time, hh:mm)	7:49 AM (1:20)
Sleep Duration (in minutes)	461.8 (54.7)
Sleep Maintenance Efficiency (percentage)	90.9% (2.8%)
MVPA (in minutes)	45.3 (25.0)
Sedentary Time (in minutes)	390.0 (91.5)

Note. MVPA is moderate-to-vigorous physical activity.

On average, our sample of adolescents had a sleep onset of 12:04 AM, a sleep offset of 7:49 AM, sleep duration of 461.8 minutes (7.7 hours; SD = 54.7 minutes), and sleep maintenance efficiency of 90.9% per night. During the daytime, they averaged 45 minutes of MVPA (SD = 25.0) and roughly 390 minutes spent in sedentary behavior (6.5 hours; SD = 91.5 minutes).

### Temporal associations of daytime physical activity predicting that night’s sleep

#### Moderate-to-vigorous physical activity

**Figure 2:**
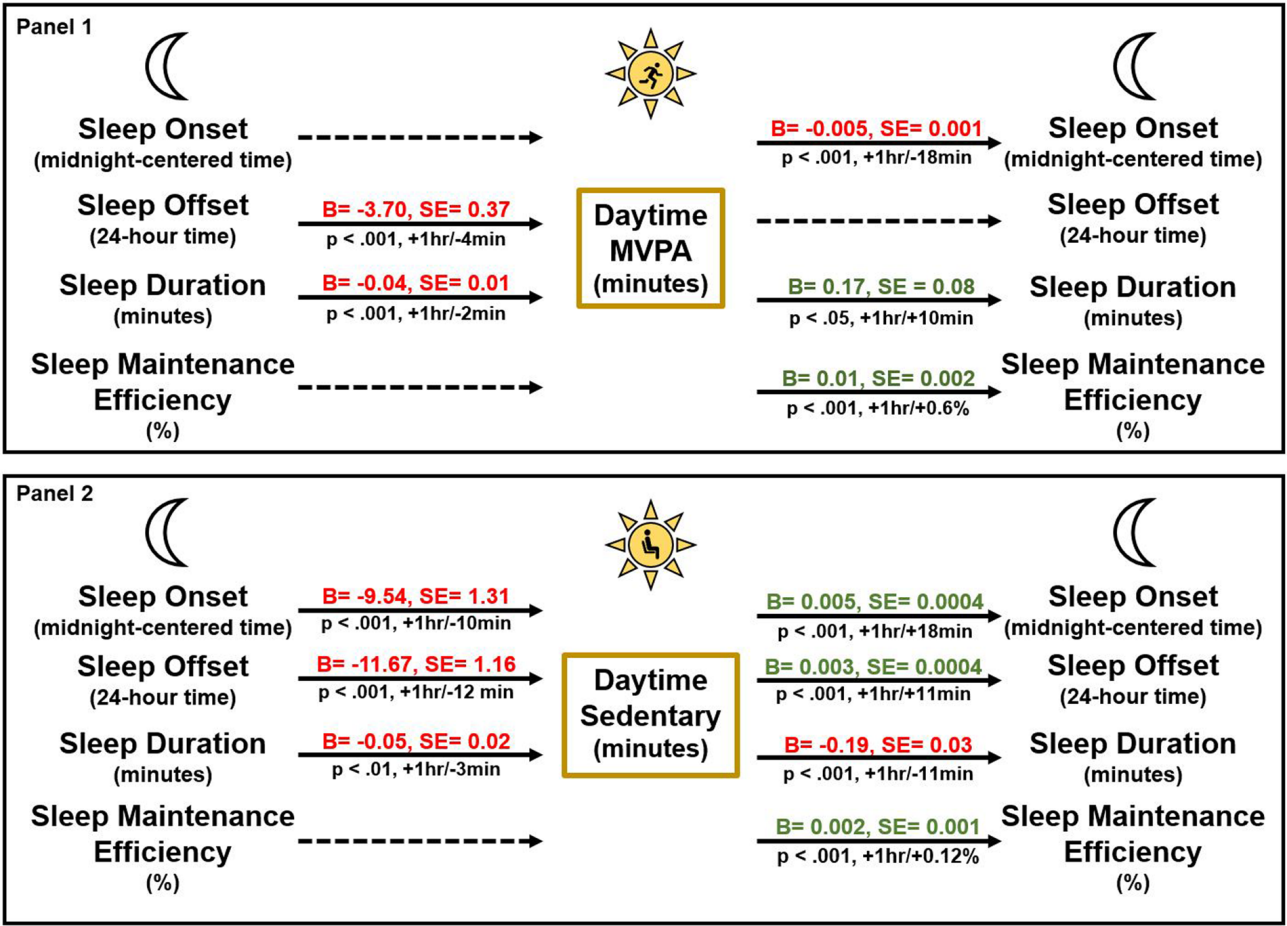
Temporal Associations between Nighttime Sleep Variables and Minutes in Daytime MVPA (panel 1) and Daytime Sedentary Behavior (panel 2). *Panel 1*: Delayed sleep offset and longer sleep duration was associated with less MVPA the next day (p < 0.001). More MVPA was associated with earlier sleep onset (p < 0.001), longer sleep duration (p < 0.05), and higher sleep maintenance efficiency (p < 0.001). *Panel 2*: Nights with earlier sleep onset (p < 0.001), earlier sleep offset (p < 0.001), and shorter sleep duration (p < 0.01) were associated with more next-day sedentary behavior. More daytime sedentary behavior was associated with later sleep onset (p < 0.001), later sleep offset (p < 0.001), shorter sleep duration (p < 0.001), and higher sleep maintenance efficiency (p < 0.001) that night. Results adjusted for covariates, including sex, age, mother’s education, family structure, household income to national poverty threshold, and age percentile body mass index. Note. MVPA is moderate-to-vigorous physical activity.

Table 2 shows results of a series of multilevel models examining the associations of daytime MVPA with nightly sleep variables. Within-person MVPA was significantly associated with sleep onset (B = −0.005, p < 0.0001), such that on days with more minutes in MVPA than their individual average, adolescents fell asleep earlier that night. A one-hour increase in MVPA was associated with 0.3 hours (18 minutes) earlier sleep onset (−0.005 * 60). Within-person MVPA was also a significant predictor of sleep duration (B = 0.17, p = 0.03), such that on days with more minutes in MVPA than their individual average, adolescents slept longer that night. A one-hour increase in MVPA was associated with 10 more minutes of sleep duration (0.17 * 60). Lastly, within-person MVPA was also a significant predictor of sleep maintenance efficiency, where adolescents who were more physically active than their average during the day had a higher sleep maintenance efficiency during their night sleep (B = 0.01, p < 0.0001). These effects were present after adjusting for sociodemographic, health and daily covariates. The within-person effects were independent of between-person associations between MVPA and sleep variables, such that those who engaged in more MVPA than others in the sample had earlier sleep onset and offset, on average, across days (no between-person associations of MVPA with sleep duration or maintenance efficiency). For within-person results of MVPA to all sleep variables, refer to Panel 1 of Fig. 2.

**Table 2:** Results from Multilevel Models of Daytime MVPA Minutes Predicting Night Sleep Variables.

	Sleep onset (midnight-centered time)		Sleep offset (24-hour time)		Sleep duration (minutes)		Sleep maintenance efficiency (percentage)	
	B	(SE)	B	(SE)	B	(SE)	B	(SE)
**Intercept**	−4.48	(1.87)*	0.68	(1.85)	410.67	(77.37)***	96.59	(3.86)***
**Sex**, **Male** (**ref Female**)	0.62	(0.14)***	0.38	(0.13)**	−16.56	(5.60)**	−0.76	(0.28)**
**Age**	0.24	(0.12)*	0.42	(0.12)***	4.01	(4.97)	−0.30	(0.25)
**Race, White, Non-Hispanic (ref)**								
Black, non-Hispanic	−0.09	(0.21)	−0.35	(0.21)	−7.80	(8.76)	−0.25	(0.44)
Hispanic	−0.05	(0.23)	0.10	(0.23)	18.50	(9.36)*	0.01	(0.47)
Other	−0.37	(0.25)	−0.43	(0.25)	1.14	(10.21)	−0.06	(0.52)
**Mother’s Education, Less than High School (ref)**								
High School or Equivalent	0.13	(0.23)	0.03	(0.22)	−7.50	(9.30)	0.12	(0.47)
Some College/Technical School	0.26	(0.20)	0.08	(0.20)	−14.74	(8.39)	0.19	(0.42)
College or Graduate School	−0.04	(0.28)	−0.04	(0.28)	2.02	(11.52)	0.84	(0.57)
**Family Structure, Biomother** + **Biofather (ref)**								
Other Living Arrangements	−0.38	(0.25)	−0.10	(0.25)	17.93	(10.34)	−0.48	(0.52)
Biomother + New Partner	−0.30	(0.19)	−0.005	(0.19)	15.65	(7.88)*	−0.97	(0.39)*
Biomother Only	−0.16	(0.18)	0.12	(0.18)	9.98	(7.48)	−0.63	(0.37)
**Income to Poverty Threshold, >=300% (ref)**								
<49%	0.57	(0.26)*	0.65	(0.25)*	8.08	(10.59)	−1.38	(0.53)**
50–99%	0.16	(0.23)	0.002	(0.23)	−6.49	(9.54)	0.25	(0.48)
100–199%	0.21	(0.20)	0.21	(0.20)	−3.52	(8.20)	−0.52	(0.41)
200–299%	−0.09	(0.23)	−0.02	(0.22)	−4.61	(9.24)	0.08	(0.47)
**BMI**	0.003	(0.003)	−0.002	(0.003)	−0.25	(0.11)*	−0.001	(0.01)
**Weekend** (**ref Weekday**)	0.93	(0.07)***	1.86	(0.08)***	54.74	(4.88)***	0.07	(0.13)
**Daytime MVPA Minutes** (**between-person**)	−0.01	(0.003)***	−0.01	(0.003)***	−0.21	(0.11)	0.01	(0.01)
**Daytime MVPA Minutes** (**within-person**)	−0.005	(0.001)***	0.001	(0.001)	0.17	(0.08)*	0.01	(0.002)***

Note. 2,249 daily observations were clustered within 417 adolescents. Results express the between- and within- relationship of daytime minutes in MVPA and night sleep variables while adjusting for covariates.

MVPA is moderate-to-vigorous physical activity. Biomother, Biofather = Biological Mother and Biological Father. BMI is body mass index.

*p < 0.05, **p < 0.01, ***p < 0.001.

#### Sedentary behavior

The results from models with sedentary behavior as the main predictor of nightly sleep variables, adjusting for covariates and between-person associations, are presented in Table 3. At the within-person level, adolescents who were more sedentary during the day fell asleep later (B = 0.005, p < 0.0001) and woke up later (B = 0.003, p < 0.0001), such that with a one-hour increase above their average in sedentary behavior during the day, sleep onset and offset were delayed by 18 and 11 minutes, respectively. In addition, time in sedentary behavior during the day was inversely related to that night’s sleep duration (B = −0.19, p < 0.0001). That is, on days when adolescents were more sedentary than their average, they slept shorter that night. Specifically, a one-hour increase in sedentary behavior was associated with an 11-minute reduction in sleep duration (−0.19 * 60). Finally, adolescents who were more sedentary during the day than their average slept more efficiently that night (B = 0.002, p = 0.0005). For within-person results of sedentary behavior to all sleep variables, refer to Panel 2 of Fig. 2. Additionally, there were between-person associations between minutes in sedentary time and sleep variables, such that those who spent more time in sedentary behavior than the sample mean had later sleep onset (p = 0.0003), shorter sleep duration (p < 0.0001), and higher sleep maintenance efficiency (p = 0.0005).

**Table 3:** Results from Multilevel Models of Daytime Sedentary Minutes Predicting Night Sleep Variables.

	Sleep onset (midnight-centered time)		Sleep offset (24-hour time)		Sleep duration (minutes)		Sleep maintenance efficiency (percentage)	
	B	(SE)	B	(SE)	B	(SE)	B	(SE)
**Intercept**	−4.02	(1.89)*	0.74	(1.93)	381.27	(75.27)***	97.63	(3.81)***
**Sex**, **Male** (**ref Female**)	0.63	(0.14)***	0.30	(0.14)*	−22.26	(5.45)***	−0.57	(0.28)*
**Age**	0.21	(0.12)	0.42	(0.12)***	6.30	(4.83)	−0.38	(0.25)
**Race, White, non-Hispanic (ref)**								
Black, non-Hispanic	−0.10	(0.21)	−0.42	(0.22)	−11.54	(8.48)	−0.16	(0.43)
Hispanic	−0.05	(0.23)	0.14	(0.23)	21.39	(9.10)*	−0.10	(0.46)
Other	−0.38	(0.25)	−0.43	(0.26)	2.63	(9.91)	−0.13	(0.51)
**Mother’s Education, Less than High School (ref)**								
High School or Equivalent	0.17	(0.23)	0.11	(0.23)	−6.06	(9.00)	0.09	(0.46)
Some College/Technical School	0.30	(0.20)	0.18	(0.21)	−11.44	(8.12)	0.08	(0.41)
College or Graduate School	0.02	(0.28)	0.08	(0.29)	5.35	(11.15)	0.74	(0.57)
**Family Structure, Biomother** + **Biofather (ref)**								
Other Living Arrangements	−0.42	(0.25)	−0.21	(0.26)	13.39	(10.00)	−0.37	(0.51)
Biomother + New Partner	−0.30	(0.19)	−0.08	(0.20)	11.04	(7.64)	−0.82	(0.39)*
Biomother Only	−0.17	(0.18)	0.08	(0.19)	7.87	(7.25)	−0.57	(0.37)
**Income to Poverty Threshold, >=300% (ref)**								
<49%	0.64	(0.26)*	0.61	(0.26)*	0.76	(10.33)	−1.15	(0.53)*
50–99%	0.23	(0.23)	0.04	(0.24)	−9.60	(9.27)	0.36	(0.47)
100–199%	0.26	(0.20)	0.25	(0.20)	−4.21	(7.95)	−0.47	(0.41)
200–299%	−0.05	(0.23)	0.05	(0.23)	−2.83	(8.95)	0.03	(0.46)
**BMI**	0.002	(0.003)	−0.003	(0.003)	−0.26	(0.11)*	−0.001	(0.01)
**Weekend** (**ref Weekday**)	0.93	(0.07)***	1.86	(0.08)***	54.48	(4.78)***	0.07	(0.13)
**Daytime Sedentary Minutes** (**between-person**)	0.003	(0.001)***	0.001	(0.001)	−0.15	(0.03)***	0.01	(0.002)***
**Daytime Sedentary Minutes** (**within-person**)	0.005	(0.0004)***	0.003	(0.0004)***	−0.19	(0.03)***	0.002	(0.001)***

Note. 2,249 daily observations were clustered within 417 adolescents. Results express the between- and within- relationship of daytime sedentary minutes and night sleep variables while adjusting for covariates. Biomother, Biofather = Biological Mother and Biological Father. BMI is body mass index.

*p < 0.05, **p < 0.01, ***p < 0.001.

#### Covariate coefficients

Significant covariates emerged in the models where daytime physical activity was predictive of sleep outcomes (Tables 2 and 3). For example, males had later sleep onset and offset, shorter sleep duration, and lower sleep maintenance efficiency than females. Adolescents who were older had a later sleep onset and offset in the MVPA to nighttime sleep models. Hispanic adolescents had longer sleep duration than white adolescents. In addition, those with higher BMI had shorter sleep duration. Finally, weekend nights (Friday and Saturday) were associated with later sleep onset and offset, and longer sleep duration, but not improved sleep maintenance efficiency.

### Exploratory analyses on timing of MVPA predicting that night’s sleep

To test if timing of physical activity is associated with sleep at the within-person level, we examined the association between the timing of the midpoint of the densest MVPA cluster on that day with sleep characteristics that night. First, we calculated the midpoint time of the largest number of minutes of MVPA within any two-hour span during the daytime hours between sleep offset and sleep onset for each day. We also quantified the number of minutes spent in MVPA within that two-hour span, which was used to quantify the average amount of MVPA that participants in our sample performed during their daily maximum MVPA cluster. We then stratified this sample to examine only individuals who averaged at least 10 minutes of MVPA during their maximum cluster (n = 293 out of our total N = 417), as previous physical activity guidelines suggest that exercise should be performed in episodes of at least 10 minutes for health benefits^13^. Finally, we centered this midpoint variable at the person mean, a metric of the change in timing of physical activity, and predicted subsequent nighttime sleep variables (sleep onset, offset, duration, maintenance efficiency). Our results show that, when the midpoint of maximum MVPA occurred earlier than an individual’s average time, sleep onset that night was slightly but significantly earlier (B = −0.019, p < 0.05), but duration, efficiency or offset timing were unchanged.

### Exploratory analyses on light intensity effects on MVPA predicting that night’s sleep

We tested two separate light level variables gathered from the wrist devices as covariates in the daytime MVPA and sedentary minutes to night sleep variable models. These variables were average white light level (lux) during the two hours with the densest MVPA cluster, and average white light level (lux) during the entire daytime portion of a participant’s day–between sleep offset and sleep onset. Neither of these variables was a significant daily covariate in any of the models where daytime MVPA or sedentary minutes predicted night sleep variables, or altered within- or between-person results (data not shown). Therefore, we did not include these measures as covariates in our models.

### Temporal associations of nighttime sleep predicting next-day’s physical activity

#### Sleep onset

Table 4 shows results of multilevel models examining the associations of nighttime sleep onset with daytime MVPA and sedentary behavior, adjusting for covariates and between-person associations. Within individuals, later sleep onset predicted less next-day sedentary behavior (B = −9.54, p < 0.0001); however, sleep onset did not significantly predict next-day MVPA. The between-person associations were significant but in the opposite direction, such that adolescents who had later sleep onset than others spent less time in MVPA (p = 0.0013) and more time in sedentary behavior (p = 0.0008) on average across days.

**Table 4:** Results from Multilevel Models of Nighttime Sleep Onset Predicting Minutes in Next-day Physical Activity (MVPA, Sedentary).

	MVPA minutes		Sedentary minutes	
	B	(SE)	B	(SE)
**Intercept**	36.99	(33.37)	258.11	(122.56)*
**Sex**, **Male** (**ref Female**)	8.00	(2.44)**	−36.50	(8.96)***
**Age**	0.33	(2.15)	9.90	(7.89)
**Race, White, non-Hispanic (ref)**				
Black, non-Hispanic	5.73	(3.75)	−8.67	(13.78)
Hispanic	−2.26	(4.03)	20.35	(14.80)
Other	−0.69	(4.44)	16.71	(16.32)
**Mother’s Education, Less than High School (ref)**				
High School or Equivalent	−4.31	(3.99)	5.29	(14.67)
Some College/Technical School	−6.42	(3.58)	16.66	(13.17)
College or Graduate School	−7.65	(4.90)	16.08	(17.99)
**Family Structure, Biomother** + **Biofather (ref)**				
Other Living Arrangements	7.62	(4.47)	−8.66	(16.46)
Biomother + New Partner	5.35	(3.38)	−16.68	(12.40)
Biomother Only	3.13	(3.20)	−4.46	(11.76)
**Income to Poverty Threshold, >=300% (ref)**				
<49%	5.70	(4.57)	−42.65	(16.80)*
50–99%	0.28	(4.10)	−21.07	(15.08)
100–199%	−2.44	(3.53)	−10.92	(12.96)
200–299%	−3.96	(4.00)	7.85	(14.73)
**BMI**	0.08	(0.05)	0.01	(0.18)
**Weekend** (**ref Weekday**)	−21.22	(1.35)***	−12.75	(4.24)**
**Midnight-centered Sleep Onset** (**between-person**)	−2.99	(0.92)**	11.46	(3.39)***
**Midnight-centered Sleep Onset** (**within-person**)	−0.39	(0.42)	−9.54	(1.31)***

Note. 2,571 daily observations were clustered within 417 adolescents. Results express the between- and within- relationship of midnight-centered sleep onset (in hours) and daytime MVPA and sedentary minutes while adjusting for covariates. Note. MVPA is moderate-to-vigorous physical activity. Biomother, Biofather = Biological Mother and Biological Father. BMI is body mass index.

*p < 0.05, **p < 0.01, ***p < 0.001.

#### Sleep offset

Table 5 displays the results of multilevel models examining the associations of nighttime sleep offset with daytime MVPA and sedentary behavior, adjusting for covariates and between-person associations. When an individual had a later sleep offset than their average, they participated in less next-day MVPA (B = −3.70, p < 0.0001) and less sedentary behavior (B = −11.67, p < 0.0001). The between person association of sleep offset and minutes in MVPA was in the same direction as the within-person results (B = −4.87, p < 0.0001).

**Table 5:** Results from Multilevel Models of Nighttime Sleep Offset Predicting Minutes in Next-day Physical Activity (MVPA, Sedentary).

	MVPA minutes		Sedentary minutes	
	B	(SE)	B	(SE)
**Intercept**	19.76	(32.83)	213.77	(125.17)
**Sex**, **Male** (**ref Female**)	8.17	(2.36)***	−30.40	(8.99)***
**Age**	1.38	(2.12)	12.26	(8.07)
**Race, White, non-Hispanic (ref)**				
Black, non-Hispanic	4.03	(3.68)	−9.48	(14.03)
Hispanic	−1.54	(3.94)	19.84	(15.04)
Other	−1.58	(4.34)	14.07	(16.58)
**Mother’s Education, Less than High School (ref)**				
High School or Equivalent	−4.17	(3.90)	6.62	(14.88)
Some College/Technical School	−6.45	(3.50)	18.53	(13.35)
College or Graduate School	−7.54	(4.79)	15.17	(18.27)
**Family Structure, Biomother** + **Biofather (ref)**				
Other Living Arrangements	7.68	(4.36)	−12.61	(16.68)
Biomother + New Partner	6.11	(3.29)	−19.62	(12.54)
Biomother Only	3.74	(3.13)	−6.55	(11.92)
**Income to Poverty Threshold, >=300% (ref)**				
<49%	7.78	(4.49)	−38.66	(17.12)*
50–99%	−0.01	(4.01)	−18.97	(15.29)
100–199%	−1.67	(3.45)	−8.93	(13.16)
200–299%	−3.19	(3.92)	8.07	(14.96)
**BMI**	0.06	(0.05)	0.03	(0.18)
**Weekend** (**ref Weekday**)	−14.37	(1.45)***	0.81	(4.56)
**Midnight-centered Sleep Offset** (**between-person**)	−4.87	(0.90)***	1.50	(3.43)
**Midnight-centered Sleep Offset** (**within-person**)	−3.70	(0.37)***	−11.67	(1.16)***

Note. 2,571 daily observations were clustered within 417 adolescents. Results express the between- and within- relationship of sleep offset (24-hour time, in hours) and daytime MVPA and sedentary minutes while adjusting for covariates. Note. MVPA is moderate-to-vigorous physical activity. Biomother, Biofather = Biological Mother and Biological Father. BMI is body mass index.

*p < 0.05, **p < 0.01, ***p < 0.001.

#### Sleep duration

The results from models with nighttime sleep duration as the main predictor of next-day MVPA and sedentary behavior, adjusting for covariates and between-person associations, are presented in Table 6. Nighttime sleep duration was significantly associated with next-day MVPA, such that on days following nights with longer sleep duration than an individual’s average, adolescents participated in less MVPA (B = −0.04, p < 0.0001). Additionally, when adolescents slept longer than their average, they were less sedentary the following day (B = −0.05, p = 0.004). The between-person associations between sleep duration and both MVPA and sedentary behavior were also significant in the same directions (p = 0.003 and p < 0.0001, respectively).

**Table 6:** Results from Multilevel Models of Nighttime Sleep Duration Predicting Minutes in Next-day Physical Activity (MVPA, Sedentary).

	MVPA minutes		Sedentary minutes	
	B	(SE)	B	(SE)
**Intercept**	51.22	(33.05)	200.81	(119.87)
**Sex**, **Male** (**ref Female**)	5.61	(2.42)*	−36.45	(8.79)***
**Age**	−0.48	(2.13)	14.03	(7.73)
**Race, White, non-Hispanic (ref)**				
Black, non-Hispanic	5.32	(3.76)	−12.47	(13.63)
Hispanic	−1.39	(4.05)	27.16	(14.70)
Other	0.15	(4.43)	13.28	(16.10)
**Mother’s Education, Less than High School (ref)**				
High School or Equivalent	−5.13	(3.99)	5.02	(14.49)
Some College/Technical School	−7.89	(3.58)*	15.21	(13.00)
College or Graduate School	−7.97	(4.90)	16.55	(17.79)
**Family Structure, Biomother** + **Biofather (ref)**				
Other Living Arrangements	9.65	(4.47)*	−8.94	(16.25)
Biomother + New Partner	7.48	(3.38)*	−14.30	(12.26)
Biomother Only	4.21	(3.20)	−3.35	(11.63)
**Income to Poverty Threshold, >=300% (ref)**				
<49%	4.69	(4.56)	−34.71	(16.55)*
50–99%	−0.79	(4.10)	−21.61	(14.90)
100–199%	−3.13	(3.52)	−9.57	(12.79)
200–299%	−4.19	(4.01)	7.19	(14.56)
**BMI**	0.06	(0.05)	−0.07	(0.17)
**Weekend** (**ref Weekday**)	−18.99	(1.31)***	−18.17	(4.14)***
**Nighttime Sleep Duration** (**between-person**)	−0.07	(0.02)**	−0.38	(0.08)***
**Nighttime Sleep Duration** (**within-person**)	−0.04	(0.01)***	−0.05	(0.02)**

Note. 2,571 daily observations were clustered within 417 adolescents. Results express the between- and within- relationship of nighttime sleep duration (in minutes) and daytime MVPA and sedentary minutes while adjusting for covariates. Note. MVPA is moderate-to-vigorous physical activity. Biomother, Biofather = Biological Mother and Biological Father. BMI is body mass index.

*p < 0.05, **p < 0.01, ***p < 0.001.

#### Sleep maintenance efficiency

Table 7 shows results of the multilevel models examining the associations of nighttime sleep maintenance efficiency with daytime MVPA and sedentary behavior, adjusting for covariates and between-person associations. Sleep maintenance efficiency did not significantly predict MVPA or minutes in sedentary time at the within-person level. However, there was a positive, between-person association between sleep maintenance efficiency and sedentary time, such that those who had higher sleep efficiency than the sample mean spent more time in sedentary behavior (p = 0.0005). For within-person results of all nighttime sleep variables to MVPA and sedentary behavior, refer to Fig. 2.

**Table 7:** Results from Multilevel Models of Nighttime Sleep Maintenance Efficiency Predicting Minutes in Next-day Physical Activity (MVPA, Sedentary).

	MVPA minutes		Sedentary minutes	
	B	(SE)	B	(SE)
**Intercept**	51.29	(33.52)	178.08	(121.56)
**Sex**, **Male** (**ref Female**)	6.75	(2.45)**	−26.32	(8.89)**
**Age**	−0.56	(2.16)	14.56	(7.83)
**Race, White, non-Hispanic (ref)**				
Black, non-Hispanic	6.23	(3.80)	−7.07	(13.79)
Hispanic	−2.29	(4.08)	20.84	(14.80)
Other	0.30	(4.48)	13.69	(16.29)
**Mother’s Education, Less than High School (ref)**				
High School or Equivalent	−4.83	(4.04)	6.42	(14.66)
Some College/Technical School	−7.25	(3.62)*	17.74	(13.15)
College or Graduate School	−8.02	(4.97)	12.14	(18.02)
**Family Structure, Biomother** + **Biofather (ref)**				
Other Living Arrangements	8.90	(4.52)*	−11.74	(16.42)
Biomother + New Partner	6.63	(3.43)	−14.54	(12.45)
Biomother Only	3.77	(3.24)	−3.68	(11.77)
**Income to Poverty Threshold, >=300% (ref)**				
<49%	4.54	(4.64)	−31.32	(16.85)
50–99%	−0.34	(4.15)	−20.09	(15.07)
100–199%	−2.94	(3.57)	−6.25	(12.97)
200–299%	−3.96	(4.06)	8.74	(14.74)
**BMI**	0.08	(0.05)	0.04	(0.18)
**Weekend** (**ref Weekday**)	−21.63	(1.29)***	−21.51	(4.03)***
**Nighttime Sleep Maintenance Efficiency** (**between-person**)	0.28	(0.45)	5.71	(1.63)***
**Nighttime Sleep Maintenance Efficiency** (**within-person**)	0.34	(0.23)	1.16	(0.71)

Note. 2,571 daily observations were clustered within 417 adolescents. Results express the between- and within- relationship of nighttime sleep maintenance efficiency (in percentage) and daytime MVPA and sedentary minutes while adjusting for covariates. Note. MVPA is moderate-to-vigorous physical activity. Biomother, Biofather = Biological Mother and Biological Father. BMI is body mass index.

*p < 0.05, **p < 0.01, ***p < 0.001.

#### Covariate coefficients

Some of the covariates in the nighttime sleep models as the main predictor were also associated with minutes in MVPA and sedentary behavior (Tables 4–7). Males (vs. females) had more MVPA and less sedentary behavior in all models. Individuals whose mothers had some college and technical school (vs. less than high school) had less MVPA overall in the sleep duration and sleep maintenance efficiency to MVPA models. Additionally, adolescents spent less time in both MVPA and sedentary time on weekends than on weekdays.

## Discussion

Physical activity and sleep are modifiable health behaviors previously reported to be related to each other; yet, less is known about how each predicts and is predicted by the other in adolescents’ naturalistic daily contexts. The current study examined the temporal associations between nightly sleep and daily physical activity during the school year in a demographically diverse sample of 417 adolescents using rigorous actigraphy methods (using separate, previously validated algorithms for each sleep and physical activity). In support of our hypotheses, results revealed that on days when adolescents were more physically active than their average, they had earlier sleep onset, longer sleep duration, and higher sleep maintenance efficiency. Conversely, when adolescents were more sedentary during the day, they had later sleep onset and shorter sleep duration, but had higher sleep maintenance efficiency. When analyzing the opposite directionality, a later sleep onset was associated with less sedentary behavior the following day. Longer sleep duration and later sleep offset were associated with less MVPA and less sedentary time the next day; however, there was no observed effect of sleep maintenance efficiency on next-day physical activity or sedentary time. These findings advance our understanding of the complex, within-person associations between sleep, physical activity, and sedentary behavior using objective measures of these behaviors, and support the development of health behavior interventions leveraging these linkages to improve health and well-being in adolescents.

Our results demonstrate that increasing minutes of moderate-to-vigorous physical activity during the day has potential to improve adolescents’ sleep health that night. Specifically, for each additional hour of MVPA above an individual’s average, sleep onset was 18 minutes earlier, sleep duration increased by 10 minutes, and sleep maintenance efficiency improved by 0.6%, indicating MVPA is beneficial in promoting earlier bedtimes, more sleep quantity, and better sleep quality in adolescents. These results are consistent with previous research reporting the associations of daytime MVPA and sedentary behavior with that night’s sleep duration in children^14^. The current study adds to the sleep health literature by demonstrating the positive associations of MVPA with more dimensions of measured sleep health, including sleep onset and sleep maintenance efficiency in adolescents. We further performed an exploratory analysis on timing of MVPA and found that when an adolescent was most physically active earlier in the day than their average time of greatest activity, their sleep onset was earlier that night. We also found that in 90% of days in our sample, adolescents spent less than four minutes in MVPA within two hours of their daily sleep onset, indicating that within our sample, exercise before sleep onset was unlikely. However, since our sleep timing variable was sleep onset and not self-reported bedtime, it is possible that exercising closer to bedtime may influence sleep onset latency. Future research could extend beyond our preliminary findings and investigate potential mechanisms relating MVPA to improved sleep, such as psychological benefits (e.g., lessened anxiety or depression), and reasoning for earlier bedtimes (e.g., tired from a long day of activity). Future work could also introduce a randomized-control intervention designed to increase daily physical activity^12^, which would provide more robust support of the potential causal effect of daily MVPA on that night’s sleep outcomes and support the use of school-based physical activity interventions. Taken together, our results suggest that increasing MVPA may improve sleep quality and extend sleep duration at the daily level in adolescents.

There are a number of theorized biological mechanisms by which regular and acute bouts of physical activity may increase sleep duration and improve sleep quality. First, participating in physical activity can help to reduce depressive and anxious symptoms^15,16^, both closely linked with insomnia symptoms^17^. Second, physical activity leads to a thermogenic effect, which is an initial increase in body temperature then followed by a decrease in temperature that promotes shorter sleep onset latency^18^ and slightly increases the amount of slow-wave sleep^19^. Finally, evening physical activity may extend the amount of time in bed on school nights by promoting a phase shift of the central circadian pacemaker to a more typical circadian phase^20^. This shift would oppose the typical later circadian phase and later bedtime preference that is in part a physiological manifestation of adolescent development^21^.

The U.S. Department of Health and Human Services states that adolescents should spend at least 60 minutes in moderate-to-vigorous physical activity each day in order to promote short- and long-term health benefits^22^. The number of adolescents ages 12–15 in the United States obtaining this amount at this intensity is low (about 8%) when measured by accelerometers^23^. While the average daily time spent in MVPA was 45 minutes, a greater proportion of adolescents in our sample reached the physical activity recommendations than what has been previously reported in nationally representative samples of adolescents, where on average, 22% participated in 60 minutes or more of MVPA each day. It may be that our urban sample relied more heavily on walking or other forms of physical activity for transportation^24^, and thus, spent more time in MVPA than adolescents in non-urban areas.

Within-person associations between nighttime sleep and next-day physical activity contradicted our expectation, in that adolescents who slept longer than their average engaged in fewer minutes of MVPA the next day, though this finding is consistent with some previous studies^25^. The less MVPA observed after longer nights of sleep may be the result of later wake times^26^ shortening the available time in the day for any activity, an interpretation also supported by the shorter sedentary time following nights with longer sleep. In fact, our results from the within-person model of later sleep offset predicting less next-day MVPA and sedentary behavior are in line with this hypothesis. Future research could determine whether time availability, motivation for exercise^27^, or other factors are at play. Prior research also found that women in midlife were less physically active on days following nights of extended sleep duration^28^, but this study was limited by rigid 24-hour day lengths. Our methodology may bypass this explanation by allowing physical activity times (daytime) to not be constrained to certain hours, meaning wake hours where physical activity could be performed were bookended by sleep periods, regardless of the day length. However, it is possible that adolescents from our sample lost potential opportunities across the day to be physically active when their sleep was longer the night before. It is also possible that this finding is attributable to longer sleep duration occurring more frequently on weekends instead of weekdays. Sleep duration has shown to be negatively impacted (shortened) on weekdays due to early school start times^7^, however, commuting to school, moving between classes, and participating in structured physical activity (i.e., physical education and after-school sports) likely contribute to increases in physical activity. Longer sleep duration on weekends followed by days with potentially fewer school-related physical activities might have contribute to the inverse relationship.

Our study has a number of strengths that increase our confidence in interpreting our results. Foremost, our study utilized a multilevel modeling design, which allowed for within-person or inter-daily analyses of sleep and physical activity. This approach provides a comparison of each individual’s sleep and physical activity throughout the week, which provides insight into the temporal directionality between physical activity on sleep and the reciprocal relationship after taking into account between-person differences. Second, most studies examining the association between physical activity and sleep used self-reported measures of sleep and physical activity^11,29^ that are prone to measurement error due to recall and social desirability bias. We used wrist- and hip-worn accelerometers to objectively measure both sleep and physical activity, respectively, which reduces the concern of such biases. Furthermore, there are a growing number of studies using accelerometers to measure sleep and physical activity^30–32^, yet to our knowledge, there are no studies using two separate actigraphy devices optimal for the measures of interest (e.g. wrist placement for sleep, hip placement for MVPA/sedentary) in adolescents’ daily naturalistic settings. Additionally, these studies are limited due to small sample size. Our study used a large, population-based sample of adolescents primarily comprised of racial/ethnic minorities and those from primarily low-income, single-parent homes in urban communities. Considering that low-income and urban neighborhoods may have deleterious effects on health behaviors such as physical inactivity, poor diet, or inadequate sleep, the present study suggests that policies promoting physical activity may also improve sleep health in socioeconomically disadvantaged adolescents. Finally, we used a validated physical activity classification algorithm to measure 1-minute bouts of MVPA, which were included in daily totals, whereas previous literature uses methodologies that only counts 10 consecutive minute bouts of MVPA towards daily totals. However, recent research shows that any accumulation of physical activity, regardless of duration, is important for lowering the risk of mortality^33^. The importance of obtaining at least 10 consecutive minutes of MVPA to be included in daily totals has also been omitted in the 2^nd^ edition of the Physical Activity Guidelines for Americans^22^.

Limitations include the study design not including across-season data for each participant. Seasons have been shown to affect physical activity patterns in places where temperatures and photoperiod changes are more dramatic^34^. Sleep can also vary with daily light exposure, as well as large photoperiod changes (the relative proportions of light and dark) across the year^35^; however, light data gathered from the wrist accelerometers do not capture angle of gaze considered appropriate for capturing light effects on circadian rhythms. Additionally, our results are from observational data; although our statistical models imply precedence with that day’s physical activity predicting sleep that night and vice versa, randomized-control intervention studies are needed to strengthen causal inferences about the sleep and physical activity relationship. Another limitation includes setting sleep periods without the assistance of a daily diary, as cohort studies have shown adolescent participants do not complete these reliably, and may introduce unnecessary bias of self-report to wrist movement data-based estimates of sleep. In a recent study, an accelerometer-based scoring algorithm was employed without diary information, and sleep onset and offset were accurately estimated against polysomnography^36^ so this limitation of setting sleep periods without a diary is minimized; furthermore, removing sedentary time during sleep (captured by the wrist sleep monitor) more accurately characterizes sedentary behavior (captured by the waist activity monitor). In the absence of a sleep diary, we have no measure of time in bed and therefore no measure of sleep latency or sleep efficiency; instead, the variable sleep maintenance efficiency was used in our analyses to capture efficiency after a participant’s sleep onset^37^.

For our activity intensity classification, we selected the method developed by Trost and colleagues^38^. We chose this method over traditional approaches that use cut-points for the classification of sedentary, light, and moderate-to-vigorous physical activity, derived by fitting the relationship between activity counts and energy expenditure. The traditional approach has been found to be inaccurate with high variability in regression lines^39^ and substantial estimation errors^40^. Trost’s classifier is more advanced because it employs a trained neural network and features extracted from activity counts in 1-second epochs. A limitation of applying Trost’s neural network to our dataset is the fact that it was trained in laboratory, not free-living conditions. However, a range of activities were included during the data training, possibly mapping the variety of activities that could be performed in real life, such as lying down and playing a computer game (sedentary), and walking, running, basketball, and aerobic dance (MVPA). In addition, since the aim of our study is not to establish normative ranges of activity, but to analyze data within our sample, the effect of possible bias between laboratory and free-living conditions should not be as significant as when comparing activity levels in data from different studies. Future research may need to strengthen the validation of this algorithm in naturalistic settings of adolescents.

The results from this study illustrate the within-person, bidirectional associations between objectively measured sleep and physical activity in a national sample of urban adolescents during school months (see Supplementary Data for results during summer months). Promoting daily MVPA may help to elicit earlier sleep onset, lengthen sleep duration, and enhance sleep maintenance efficiency, critical for healthy adolescent development. In turn, promoting adequate sleep may help to support more regular physical activity, in contrast to the reduced energy and lower likelihood of engaging in activity when sleep deprived^27,41,42^. Future interventions may wish to leverage the mutual associations of these essential health behaviors to help improve health and well-being in adolescents.

## Methods

### Study participants

Data were collected within the Fragile Families & Child Wellbeing Study (FFCWS; www.fragilefamilies.princeton.edu), a longitudinal study following a birth cohort of children born between 1998 and 2000 in 20 U.S. cities. By design, this study oversampled unmarried mothers and included a large proportion of racial/ethnic minority, low-income, and less-educated mothers. Procedures were approved by the Princeton and Stony Brook Universities’ Institutional Review Boards and conducted in accordance with all relevant policies, including obtaining informed consent [from parent/guardian] and assent [from child/teen]. During the Age 15 Wave of the FFCWS, when the cohort was approximately 15 years old, a randomly selected sub-sample (N = 1,090) was asked to concurrently wear an accelerometer on their non-dominant wrist and a physical activity monitor on their hip for seven consecutive days (Fig. 1). Of 1,049 assenting adolescents, 650 provided at least three valid consecutive days of sleep data with at least three valid days of physical activity data and covariate data collected from age-15 surveys. Finally, 417 of these adolescents provided data during the school year, defined by participants who had their first valid day of actigraphy in the months of September–May.

### Actigraphic sleep measures

Sleep measures were collected with a wrist-worn accelerometer (Actiwatch Spectrum; Philips-Respironics, Murrysville, PA) worn on the non-dominant wrist for one week. Wrist movement data were downloaded with Philips Actiware software (v6.0.4). At least two independent scorers (blinded to each other) determined “day” cut-point times, validity of days, and set sleep intervals using a previously validated algorithm^43^, without using information from a sleep diary. The scorers adjudicated each recording for inter-rater agreement by verifying number of valid days, cut-point, number of sleep intervals, and differences greater than 15 minutes in duration and wake-after-sleep-onset for each sleep interval. Specifically, trained scorers determined sleep intervals using a decrease in activity levels and the aid of light levels for sleep onset and sleep offset^44^, and a nighttime sleep interval was split into two intervals (main sleep and nap) if there was an awakening ≥1 hour during this interval. A sleep actigraphy day was determined invalid and no sleep interval was set if there were ≥4 total hours of off-wrist time, with the exception of the first and last day (device should be worn at least 2 hours before sleep onset on the first day), constant false activity due to battery failure, data unable to be recovered, or an off-wrist period of ≥60 minutes within 10 minutes of the scored beginning or end of the main sleep period for that day.

*Sleep onset* was determined by the scored actigraphic nighttime sleep duration start time: the time of the last 30-second epoch of activity >10 counts followed by 5 consecutive epochs ≤10, indicating sleep.

*Sleep offset* was determined by the scored actigraphic nighttime sleep duration end time: the time of the first 30-second epoch of activity >10 counts that follows 5 consecutive epochs ≤10.

*Nighttime sleep duration* was calculated by the number of minutes between sleep onset and sleep offset during the sleep interval with the longest duration between the hours of 10 PM and 8 AM in a 24-hour cut-point day.

*Nighttime sleep maintenance efficiency* was defined as the minutes of actual sleep between sleep onset and sleep offset divided by the nighttime sleep duration interval (%)^45^.

### Actigraphic physical activity measures

Physical activity measures were collected with a hip-worn tri-axial accelerometer on an elastic belt (Actigraph GT3X, Actigraph, Pensacola, FL) at a rate of 80 Hz. The data from the hip device were downloaded in Actilife software (v6.13.3, Actigraph, Pensacola, FL, USA). Wear time was determined by a validated algorithm^46^ built into the Actilife software which employs activity counts derived from acceleration. A non-wear period was defined as a minimum length of 90 minutes with consecutive zero counts and the allowance of 2-minute intervals of nonzero counts surrounded by 30-minute consecutive zero count windows for artifactual movement detection^46^. We extracted features (10^th^, 25^th^, 50^th^, 75^th^, 90^th^ percentiles, lag one autocorrelation) from 60-second windows using MATLAB R2017a software (The MathWorks, Inc., Natick, MA) using the activity counts on the device’s vertical axis exported from Actilife calculated over 1-s epochs. Using R version 3.4.0 (R Foundation for Statistical Computing, Vienna, Austria), a validated artificial neural network algorithm^38^ predicted physical activity type for each minute of every recording. Minutes of non-wear^46^ were removed from the processed data. Activity by minutes was categorized into five different groups: sedentary, light (e.g., household tasks, baseball catch), moderate-to-vigorous (MVPA), walking, and running. In this study, we focused on the sedentary and collapsed MVPA categories (moderate-to-vigorous, walking, running), given their associations with sleep in previous studies with other populations and different methodologies^14,47^. We did not evaluate minutes within the light intensity physical activity category.

#### *Moderate-to-vigorous physical activity (MVPA)*

 Activity measures included daily minutes in daytime MVPA. As the neural network we used^38^ had been trained to detect the “walking” and “running” categories using data with a minimum of 3.8 METS, and this level of energy expenditure falls within the range for MVPA for adolescents^48^, our measure of MVPA included minutes in moderate-to-vigorous-intensity games or sports, walking, and running categories.

#### *Sedentary behavior*

We assessed daily minutes in sedentary behavior, excluding daytime napping from sleep actigraphy. Example sedentary behaviors include sitting down and playing a computer game, typically less than 1.5 METS^38^.

### Covariates

We adjusted for several sociodemographic and family characteristics linked with adolescent sleep and physical activity behavior^49–51^, including sex (male vs. female), age, race (Black, Hispanic, other or missing, vs. White), mother’s education (college or graduate school, high school or equivalent, some college, vs. less than high school), family structure (biological mother, biological mother with new partner, biological father only or with new partner or other primary caregiver, vs. biological mother and biological father), and household income to national poverty threshold (<49%, 50–99%, 100–199%, 200–299%, vs. >300%). We also adjusted for body mass index measured during the home visit (BMI; age percentile). With the exception of sex (determined at birth), all covariates were measured at the age 15 wave. We further included weekend (vs. weekday) as a daily covariate in our analyses.

### Temporal alignment of sleep and physical activity measures

Sleep and physical activity data were merged together by participant ID and date/time. A “day” was defined as the time period between a participant’s nighttime sleep onset to the next day’s main/nighttime sleep onset. While each day was roughly 24 hours, this depended on the participant’s sleep schedule. For example, if a participant’s sleep onset was at 10:00 PM one night and 11:30 PM the next night, the length of that day would be 25.5 hours. A previous night’s sleep and the following day’s physical activity were aligned on the same observation record. If there was no main sleep interval at the end of a day due to an invalid sleep period (i.e., device removed), the day ended when it encountered more than 1 hour of off-wrist time or an apparent sleep interval scored by a validated algorithm^43^, whichever arrived first. The proportion of hip device wear-time in the wake portion of a day was calculated and the physical activity day was named invalid if there was >25% non-wear minutes. The wake portion of a day was also considered invalid if there was <20 hours within the day that was “forced-ended” due to an invalid sleep period. The “sedentary behavior” variable was created from a one-minute level data merge between the sleep and physical activity data, where minutes that were scored as sleep during the day (naps) did not count towards total minutes classified as sedentary time by the physical activity algorithm.

### Statistical analyses

We used multilevel models with lagged effects in SAS 9.4 software (SAS Institute, Cary, North Carolina) to test the temporal associations between nightly sleep and daily physical activity variables. Two-level models were used such that 2,571 daily observations in the nighttime sleep to next-day physical activity models and 2,249 daily observations in the daily physical activity to nighttime sleep models (smaller observations by using one-day lagged physical activity variables) were clustered within 417 adolescents. The models used an AR(1) error covariance structure to consider the fact that consecutive sleep and physical activity observations might be correlated more highly than non-consecutive observations^52^. When the outcome variable was daytime physical activity (MVPA, sedentary), either nighttime sleep duration, sleep maintenance efficiency, sleep onset, or sleep offset was the predictor variable. When the outcome variable was a nighttime sleep measure (e.g., duration, sleep maintenance efficiency, sleep onset, sleep offset), either daytime MVPA or sedentary behavior was the predictor variable. This totaled to sixteen different models (eight predicting sleep measures, eight predicting physical activity measures). Variances for sleep and physical activity measures were decomposed to within-person (level-1) and between-person (level-2) levels. Within-person variables were centered at the person mean, such that positive values indicated scores higher than the person’s own cross-day average. Between-person variables were centered at the sample mean, such that positive values indicated higher scores than others in the sample. Covariates were included in all models.

## Supplementary information

Supplementary analyses in the summer months

## Data Availability

Survey data from the Fragile Families and Child Wellbeing study (https://fragilefamilies.princeton.edu/documentation) are publicly available from Princeton University’s Office of Population Research (OPR) data archive: https://opr.princeton.edu/archive/restricted/Default.aspx. The sleep and physical activity actigraphy data sets generated and analyzed during the current study are not publicly available yet, but will be available through an application process at the above link.
