# Supplementary material for: Bidirectional, Daily Temporal Associations between Sleep and Physical Activity in Adolescents
Source: Sci Rep. 2019 May 22;9:7732. doi: 10.1038/s41598-019-44059-9 (PMC6531611; doi:10.1038/s41598-019-44059-9)
Supplement: Supplementary file 1 — Supplementary analyses in the summer months [file 41598_2019_44059_MOESM1_ESM.docx]

**Bidirectional, Daily Temporal Associations**

**between Sleep and Physical Activity in Adolescents**

Lindsay Master, Russell T. Nye, Soomi Lee, Nicole G. Nahmod,

Sara Mariani, Lauren Hale, Orfeu M. Buxton

**Supplementary Data**

***Supplementary analyses in the summer months*** Additional within-person analyses were performed on the sample of adolescents who provided sleep and physical activity actigraphy data during the summer months (June through August, n=233) and were similar to school-year results. More sedentary time than an adolescent’s average was associated with shorter sleep duration (B=-0.19, p<.0001), later sleep onset (B=0.008, p<.0001) and later sleep offset (B=0.005, p<.0001) that night. Nights with longer than an adolescent’s typical sleep duration were followed by days with less time in both MVPA (B=0.03, p<.0001) and sedentary behavior (B=-0.07, p=.004). A later sleep onset than an adolescent’s average predicted less time in sedentary behavior the following day (B=-8.34, p<.0001), and a later sleep offset indicated less minutes in MVPA (B=-2.54, p<.0001) and sedentary behavior (B=-13.25, p<.0001) the following day. There was no association between daytime MVPA and that night’s sleep variables at the within-person level, indicating that summer schedules likely contribute to changes in physical activity and sleep behavior.
